# Bone Mineral Density, Body Composition, and Metabolic Health of Very Low Birth Weight Infants Fed in Hospital Following Current Macronutrient Recommendations during the First 3 Years of Life

**DOI:** 10.3390/nu13031005

**Published:** 2021-03-20

**Authors:** Walter Mihatsch, Izaskun Dorronsoro Martín, Vicente Barrios-Sabador, María L. Couce, Gabriel Á. Martos-Moreno, Jesús Argente, José Quero, Miguel Saenz de Pipaon

**Affiliations:** 1 Department of Pediatrics, Ulm University and Neu-Ulm University of Applied Sciences, 89231 Neu-Ulm, Germany; Walter.mihatsch@uni-ulm.de; 2 Department of Neonatology, Hospital Universitario La Paz, Department of Pediatrics, Universidad Autonoma de Madrid, 28046 Madrid, Spain; izaskun.dorronsoro@gmail.com (I.D.M.); josequeroj@gmail.com (J.Q.); 3 Department of Endocrinology, Hospital Infantil Universitario Niño Jesús, Instituto de Investigación La Princesa, Department of Pediatrics, Universidad Autónoma de Madrid, 28039 Madrid, Spain; vicente.barriossa@salud.madrid.org (V.B.-S.); gabrielangelmartos@yahoo.es (G.Á.M.-M.); jesus.argente@fundacionendo.org (J.A.); 4 CIBER Fisiopatología de la obesidad y nutrición (CIBERobn), Instituto de Salud Carlos III, 28029 Madrid, Spain; 5 Neonatology Department, University Clinical Hospital of Santiago de Compostela, IDIS-Health Research Institute of Santiago de Compostela, Universidad de Santiago de Compostela, 15704 Santiago de Compostela, Spain; maria.luz.couce.pico@sergas.es; 6 IMDEA Institute, 28049 Madrid, Spain

**Keywords:** body composition, dual-energy X-ray absorptiometry, outcome, preterm infant, very low birth weight

## Abstract

The present study longitudinally evaluated growth, bone mineral density, body composition, and metabolic health outcome in very low birth weight (VLBW) infants whose in-hospital target nutrient intake was within recent recommendations. From six months to three years, bone mineral density (dual-energy X-ray absorptiometry, DXA), body composition, and metabolic health outcome were compared with a reference group of term infants. The aim was to test whether in-hospital achieved weight gain until 36 weeks of gestation (light or appropriate for term equivalent age; LTEA or ATEA) predicts later growth, bone mineral density (BMD), abdominal obesity, or metabolic health outcomes such as insulin resistance, relative to term infants, during the first three years of life. Target in-hospital energy and protein intake was not achieved. Growth in weight, length and head circumference, mid arm circumference, adiposity, fat free mass (FFM), and bone mineralization in VLBW infants was less than those in term infants and influenced by nutritional status at discharge. Preterm infants had poorer motor and cognitive outcomes. Post-discharge body composition patterns indicate FFM proportional to height but lower fat mass index in LTEA preterm infants than term infants, with no evidence of increased truncal fat in preterm infants. The hypothesis of early BMD catch-up in VLBW infants after discharge was not supported by the present data. The clinical significance of these findings is unclear. The data may suggest a reduced obesity risk but an increased osteoporosis risk. Since postnatal growth restriction may have permanent negative health effects, LTEA VLBW infants would especially appear to benefit from targeted preventive interventions. Further follow-up of the infants is required.

## 1. Introduction

The goal for the nutritional care in preterm infants is to achieve intra-uterine growth and body composition to obtain a functional outcome similar to term infants [1]. This concept is supported by the observed associations between postnatal nutritional deficits and/or growth failure and long-term neurodevelopmental impairment or metabolic syndrome surrogate markers such as higher blood pressure, abdominal obesity, and impaired glucose tolerance [2,3,4,5]. Updating of recommendations requires meticulous assessment of outcome in growth, body composition, bone mineral density, and metabolic health outcome. International recommendations for parenteral and enteral nutrition are regularly updated [6,7]. Very low birth weight (VLBW) infants frequently experience suboptimal nutrient intake and exhibit growth failure within the neonatal intensive care units (NICU) [2]. In addition, disease severity and postnatal extracellular volume contraction have been suggested to contribute to postnatal growth failure [8,9]. Suboptimal nutrient intake may permanently affect later cognitive attainment [2]. On the other hand, more recent data suggest that with improved nutritional care early postnatal growth failure in preterm infants is inevitable [8,10,11]. However, at the time of discharge from the hospital or at term equivalent age (TEA), a considerable percentage of VLBW infants still appear growth retarded (light for TEA (LTEA) defined by a weight standard deviation (SD) score at 36 weeks of gestation less than -2 SD) [12]. Given the improvement in nutritional care, it is even more important to understand the relationship between being discharged LTEA and long-term outcomes in this population [10].

Several aspects of body composition, in particular the amount and distribution of body fat, are now understood to be important health outcomes in infants and children, and dual-energy X-ray absorptiometry (DXA) remains the gold standard for body composition assessment [13]. The body composition at TEA of infants born preterm is reported to be different from that of infants born at term. Less lean tissue but similar fat mass (FM) have been found [14]. On the other hand, fat mass index (FMI) has been reported to be significantly lower in preterm infants at 8–12 years of age [15] in contrast to more total fat mass in young adults after preterm birth [16]. Therefore, there is a need to determine whether improved in hospital nutritional management can enhance lean tissue acquisition. Hence, a need for measuring body composition in the clinical management of these patients has been proposed [14].

Low birth weight (LBW) is associated with an increased risk of insulin resistance, truncal accumulation of fat, metabolic syndrome, and cardiovascular disease in adulthood [17,18]. Likewise, in preterm infants, weight gain before discharge and during the first 3 months post-discharge weight gain have been related to later metabolic disease or obesity [19,20]. On the other hand, in adolescents born preterm, weight gain during childhood is reported to be strongly associated with adverse metabolic outcome but not pre-discharge or early post-discharge growth [21]. Notably, the effect of birth weight on insulin resistance and later cardiovascular disease (CVD) is most apparent in the upper tercile of normal BMI and in obese subjects. The greatest variation in rates of weight gain is seen in the first two years of life when infants may show significant “catch-up” or “catch-down” growth. At the opposite extreme, the effect of high birth weight on later insulin resistance and cardiovascular disease is less clear [18]. These studies suggest that there may be an optimal range for healthy growth. Although, preterm birth is strongly associated with some components of metabolic syndrome and cardiovascular disease in adult life [22], postnatal programming of body composition and metabolic health outcomes in preterm infants has been sparsely examined. Several short-term studies suggest percentage of fat mass (FM%) catch up or recovery of growth and body composition within the first 6 months after discharge [23]. However, the long-term effects of postnatal growth failure on body composition and metabolic health outcomes have not been studied sufficiently.

Body fat distribution (trunk-abdominal level) has an impact on health as well. Truncal fat is the major component of body fat associated with metabolic diseases, including insulin resistance, type 2 diabetes, hypertension, and dyslipidemia, leading to increased risk of CVD or metabolic syndrome [24]. Abdominal adiposity can be reliably assessed by DXA [25].

White adipose tissue is an active player in the regulation of energy balance and metabolic homeostasis. It expresses receptors for most hormonal and neurological signals, and it synthesizes a wide variety of peptides that have both paracrine and endocrine actions. For example, circulating resistin, mainly produced by mononuclear cells in the adipose tissue matrix, is positively associated with interleukin 6 (IL-6) levels [26]. Numerous other factors and hormones are involved in metabolism and growth and could be modulated by the early nutritional environment. The insulin-like growth factor (IGF) system, and in particular IGF-1, has an important role in the regulation, differentiation, and proliferation of many cell types, acting as a major growth regulator in humans. Ghrelin, an enteric hormone with potent appetite stimulating effects, also stimulates growth hormone (GH) release [27]. Adults born preterm were shown to be less insulin sensitive than those born at term as assessed by hyperglycemic clamps. The leptin/soluble leptin receptor (sOB-R) system plays a key role in energy homeostasis [5]. The sOB-R is the main leptin binding protein, and the ratio of serum leptin to sOB-R provides a measure of the free leptin index, which may be a more accurate determinant of leptin function [28].

Finally, being born preterm or VLBW is associated with significant motor impairment persisting throughout childhood [29]. Growth from birth to discharge seemed to be associated with long-term motor development [30].

In the present study, target nutrient intake was within the recent European Society of Paediatric Gastroenterology, Hepatology and Nutrition (ESPGHAN) recommendations [6,7]. We hypothesized that VLBW infants who were appropriate for TEA (ATEA; defined by a SD weight score > −2 at 36 weeks) near discharge will have higher growth rates, lean body mass and fat mass, skeletal mineral deposition, and neurodevelopmental scores throughout the first three years of life than LTEA preterm infants. Thus, the objectives of this study were as follows:

(a) To longitudinally evaluate growth, body composition, bone mineral density, and metabolic health outcomes from six months to three years of life in a prospective cohort of VLBW infants starting at 36 weeks postmenstrual age, relative to term infants.
(b) To test in these infants whether growth status near discharge (LTEA vs. ATEA) predicts later growth retardation, bone mineral density, abdominal obesity, insulin resistance, or other associated metabolic health outcomes, relative to term infants, during the first three years of life.
(c) To analyze whether growth status near discharge (LTEA vs. ATEA) still predicts neurocognitive and motor-developmental outcome at two years of age.

## 2. Materials and Methods

### 2.1. Study Design and Population

A prospective, longitudinal, cohort study on growth and body composition in preterm and term infants from 6 to 36 months corrected age was performed. Infants were recruited from the Department of Neonatology at La Paz University Hospital. Among all consecutively born infants less than 34 weeks of gestation and less than 1500 g, 94 were considered eligible. Preterm infants were classified at TEA age as LTEA (*n* = 55, body weight (BW) z-score < −2 SD, according to Alexander growth charts [31]) or ATEA (*n* = 39, BW z-score ≥ −2 SD). A group of healthy, full-term infants (*n* = 34) was randomly recruited from the maternity wards as a reference group. Exclusion criteria were congenital diseases, chromosomal abnormalities, short bowel syndrome, or other digestive disorders in which absorption of nutrients was impaired. The study was approved by the local research ethics committee, and written informed parental consent was obtained.

The infants, together with an accompanying parent, were admitted to the Neonatology outpatient clinic at La Paz University Hospital in Madrid, Spain, between 08:00 and 09:00, at 6, 12, 18, 24, and 36 months of corrected age. To complete all necessary measurements, the infants usually stayed for 4–5 h. Overnight fasting blood samples were obtained between 08:30 and 09:30 h.

### 2.2. Anthropometry and Analytical Measurements

Weight, length, head circumference, upper arm circumference, and skinfold thicknesses (biceps, triceps, subscapular, suprailiac) were measured in duplicate, averaged, and recorded in every visit by 1 of 3 trained observers. Weight was measured accurate to the nearest 10 g (Seca electronic infant scale, Seca 375, Hamburg, Germany). Length was measured accurate to the nearest 0.1 cm (Seca infant length board, Seca 210, Hamburg, Germany). Skinfolds were measured using a Holtain caliper (Holtain Ltd., Crosswell, Crymych, Pembs, UK.). Circumferences were measured to the nearest mm by flexible measuring tapes.

After overnight fasting, blood samples were obtained between 08:00 h and 09:00 h, centrifuged at 4 °C, and the serum was stored at −80 °C until assayed. Serum glucose, total cholesterol, ferritin, and triglycerides were immediately quantified by enzymatic methods (autoanalyzer). Commercial radioimmunoassay (RIA) kits were used for measurement of the serum concentrations of adiponectin, acylated and total ghrelin and leptin (Linco, Inc., St. Louis, MO, USA). Resistin was measured by an enzyme-linked immunosorbent assay (ELISA) from Merck Millipore (Billerica, MA, USA). Insulin was determined by RIA (Diagnostic Products Corporation, Los Angeles, CA, USA), IGFBP-1 was measured by ELISA (Medix Biochemica, Kauniainen, Finland), and IGF-1 and IGFBP-3 by RIA (Mediagnost, Tübingen, Germany). IL-6 concentrations were determined by ultrasensitive ELISA kits from R&D Systems (Minneapolis, MN, USA) and sOB-R levels by ELISA (Bio-Vendor, Brno, Czech Republic). In all cases intra- and inter-assay coefficients of variations were lower than 10%. HOMA index was calculated as follows: HOMA index = plasma glucose (mmol/L) * serum insulin level (mU/mL)/22.5.

### 2.3. Dual-Energy X-ray Absorptiometry

Child DXA scans were performed at 6, 12, 18, 24, and 36 months (Lunar-DPX-MD; GE Healthcare, Chalfont St. Giles, UK). Scans were analyzed by using infant whole-body analysis software (GE Healthcare, Chalfont St. Giles, UK). All DXA scans were performed with the same device and software. Previously reported precision values for DXA are <1% for lean mass and <2% for fat mass (FM) in adults [15]. During scanning, infants were laying supine, wearing only a disposable diaper, swaddled in a light cotton blanket, and without sedation. Scans were deemed invalid in the case of excessive movements, and the infants were not re-scanned. Whole-body composition (total fat and lean mass) and regional (truncal) composition, whole-body bone mineral content (BMC), and bone mineral density (BMD) were measured. FM and fat free mass (FFM) were normalized for body weight and for height to give FM%, fat free mass percentage (FFM%), FMI, and fat-free mass index (FFMI), respectively. The percentage of truncal fat (PTF) was calculated using the following formula: PTF = total truncal fat/total fat × 100.

### 2.4. Clinical Characteristics

Data on clinical characteristics such as the score for neonatal acute physiology (SNAP), patent ductus arteriosus (PDA), late onset sepsis (LOS), chronic lung disease, Bell stage ≥2 necrotizing enterocolitis (NEC), periventricular leukomalacia (PVL), and retinopathy of prematurity (ROP) were extracted from the medical records (data not shown). A PDA was recorded if hemodynamically significant based on clinical an echocardiographic criteria. Chronic lung disease was defined as oxygen dependence at 36 weeks postmenstrual age. LOS was defined as clinical signs of septicemia beyond 72 h of age together with a positive blood culture. Only NEC ≥ grade 2, as defined by Bell et al. [32], was considered. Volpe classification system was used for classification of intraventricular hemorrhage [33]. PVL was graded and defined according to de Vries et al. [34]. ROP was graded according to the international classification system [35]. Illness severity and mortality risk scores were calculated at 24 h (score for neonatal acute physiology, SNAP) and 28 days (SNAP-II) [36,37].

### 2.5. Neurodevelopment Outcome

At 2 years, corrected age neurodevelopmental outcome was assessed by Bayley Scales of Infant Development (2nd edition). These assessments were performed and scored by a certified examiner who was masked to neonatal treatments and complications.

### 2.6. Statistical Analysis

Descriptive data are presented as the mean ± SD. SPSS version 9.0 was used for analyzing the data. In preterm infants age in months refers to corrected age. Student’s *t* test was used to compare groups (level of significance *p* = 0.05). Mixed model linear analyses, for growth, body composition, and biochemical indices, were performed. Group effect, time effect, and interaction between group and time were analyzed using likelihood ratio tests (LRTs). Individuals were included as random effect into the model to account for repeated measurements. Wald statistic *p* values are given in the tables.

General linear logistic regression models were used to test for differences in motor and mental development between LTEA and ATEA infants after adjusting for sex and potential confounding factors (SNAP score, SNAP-II score, PDA, ROP, late onset sepsis, chronic lung disease, NEC, and PVL).

## 3. Results

### 3.1. Characteristics of the Study Population

Anthropometric data at birth in all infants and at TEA in preterm infants are given in Table 1. In term infants at birth weight, length, head circumference, and gestational age were higher than in preterm infants (*p* < 0.001). Weight z score at birth was significantly lower in LTEA infants than in term and ATEA infants. At TEA weight, length, head circumference, weight z score, and arm circumference were significantly different between LTEA and ATEA infants.

The clinical biochemistries at TEA in LTEA and ATEA infants are given in Table 2. Fasting insulin and HOMA levels were significantly (*p* < 0.05) lower in LTEA infants. Thyroid function was investigated at the beginning of the study in every patient and found to be normal.

### 3.2. Anthropometric Evolution

Anthropometric data from 6 to 36 months of life are given in Table 3. In VLBW infants weight at TEA predicts growth and skinfold thicknesses during the first three years of life. Anthropometric indices were consistently higher in term infants than in preterm infants. In term infants and ATEA infants, a similar growth pattern for head circumference was seen. In LTEA infants head circumference was significantly smaller in the first three years of life than in the other groups. LTEA infants had significantly lower subscapular and bicipital skinfold thickness than term infants. There was no significant difference with regard to suprailiac skinfold; however, the three groups differed in tricipital skinfold thickness and mid-arm circumference over time.

### 3.3. Body Composition and Bone Mineral Density

The time course of body composition analysis by DXA is presented in Table 4. With growth, absolute amount of total FFM and FM increased gradually during the first three years of life (significant time effects, *p* < 0.001). There were significant group and interaction group x time effects showing at least that FM and FFM were significantly lower in LTEA infants than in term infants at all time points. In contrast, there were no significant time effects but significant group and interaction group x time effects with regard to FM(%) and FFM(%), showing at least that FM(%) was always lower and FFM(%) always higher in LTEA infants than in term infants. Most importantly, there were significant time, group, and interaction group x time effects with regard to FMI, showing that FMI decreased in all groups with time but was always lowest in LTEA infants. FFMI decreased with time in all groups (significant time effect), and there was no significant difference between the groups (no group and no interaction group x time effect).

At 6 months of age ATEA infants, in contrast to term infants, were significantly leaner (less FM, less FM%, less FMI). From that time on ATEA infants were, as a group, always between term and LTEA infants; however, the study was underpowered to prove significance.

With regard to TF there were significant time, group, and group x time effects, indicating that TF increased over time throughout the study period (*p* < 0.001). TF was always significantly higher in term than in LTEA infants and in addition at 6 months also significantly higher in term than ATEA infants (*p* < 0.001). However, after appropriate adjustment for total fat mass, the group effect and the group x time effect disappeared. PTF varied over time and was lower at 12, 18, and 24 months than at 6 and 36 months, but there was virtually no difference between the 3 groups.

Significant time, group, and group x time effects were observed in whole-body BMC. The BMC was significantly lower in preterm than in term infants at nearly all points of time (exception at 12 months in ATEA infants). After appropriate adjustment for length (BMC/length^2^), an interaction between group and time was still present. BMC/length^2^ significantly increased over time and was significantly lower in LTEA than in term infants. BMD also significantly increased over time (*p* < 0.001) and was at least in LTEA infants significantly lower than in term infant for the whole study period. With regard to ATEA infants, BMC, BMD, and BMC/length^2^ were significantly lower than in term infants at 6 months of age. For the rest of the study time bone mineralization measurements in ATEA infants were between the two other groups but more similar to LTEA infants.

### 3.4. Metabolic Outcome

A group effect was shown in serum IGF-1 (Table 5, *p* < 0.05). LTEA had higher serum IGF-1 levels than term infants. Serum IGFBP-1 changed over time (*p* < 0.05). Higher serum IGFBP-1 values were found at 12 months. Serum IGFBP-3 levels changed over time, with an increase at 36 months. No differences were found between the three groups in total or acyled ghrelin.

An interaction between group and time was observed in serum leptin levels. ATEA infants had significantly higher leptin than LTEA infants at 6 and 12 months. There was a significant effect of time on leptin and sOB-R. sOB-R did not differ between groups. LTEA infants had significantly lower levels of free leptin index (leptin/sOB-R) than term infants. Serum adiponectin levels showed an interaction between group and time. Resistin levels changed with group (*p* < 0.05) and with time (*p* < 0.05). LTEA infants had higher serum resistin compared with ATEA and term infants. Values of resistin increased from 6 to 12 months and remained stable thereafter.

From 6 to 36 months HOMA and fasting insulin changed with time (*p* < 0.001), but there was no difference between the groups. An effect of group (*p* < 0.01) and time (*p* < 0.01) was observed for cortisol levels. LTEA infants had lower serum cortisol levels compared with ATEA and term infants, and at three years there was no difference. Circulating IL-6 levels remained unchanged with time, during the first three years of life. No differences were found between the groups in cholesterol, triglycerides, and plasma glucose.

Ferritin levels (data not shown) were higher in term than preterm infants and higher at 6 months compared with later on. There was no significant interaction between group and/or time.

### 3.5. Neurodevelopmental Outcome

LTEA infants had lower mean Psychomotor Developmental Index PDI (83.7 ± 16.2 vs. 105.4 ± 15.2, *p* < 0.001) and lower mean Mental Developmental Index MDI (93.3 ± 26.8 vs.108.3 ± 14.1, *p* < 0.001) than term infants at two years corrected age. In ATEA infants mean PDI (90.9 ± 11 vs. 105.4 ± 15.2, *p* < 0.001) and mean MDI (93.8 ± 14 vs.108.3 ± 14.1, *p* < 0.001) were also lower than in term infants at two years corrected age. No significant differences were found between LTEA and ATEA preterm infants in PDI (*p* = 0.062) and MDI (*p* = 0.93), respectively.

## 4. Discussion

This study was designed primarily to compare growth, development of body composition, biochemical metabolic health outcomes, as well as the neurodevelopmental outcome of healthy term infants with that of VLBW preterm infants with protein and energy intakes close to recommended target intake, within the first 3 years of life. Special consideration was given to the preterm infants growth status at TEA (LTEA vs. ATEA).

This contemporary, longitudinal, prospective cohort study showed that weight at TEA significantly predicted growth, body composition, and biochemical metabolic health outcome, suggesting a programming effect.

### 4.1. Growth

The present data suggest that body weight near discharge significantly predicts long-term growth and body composition development in VLBW infants at least for the first three years of life. Several other studies previously reported early postnatal growth retardation in preterm infants. The present study adds that this effect is even more pronounced in VLBW infants who were LTEA near discharge. The weight SD score decreased during hospitalization on average by 1.14 in ATEA infants and by 1.41 in LTEA infants from birth to 36 weeks (Table 1). Our target daily intakes for energy and protein for these VLBW infants were within the recommended range of recent recommendations [6,7]. However, within the first three weeks of life an average of 95.5% for energy and 86.4% for protein of the target intake were achieved only [8]. Therefore, even though energy and protein intake were close to recommended levels, compared to term infants, fetal growth was not matched, indicating that for the group of VLBW infants nutrient intake was inadequate.

In both groups of VLBW infants, weight, length, HC, mid arm circumference, and subscapular skinfold thickness were significantly smaller than in term infants throughout the first 3 years of life (Table 1), and there was no catch-up. These data suggest a postnatal in hospital programming effect because the majority (92.3%) of the VLBW infants were appropriate for gestational age (AGA) at birth (weigh SD score > −2). We hypothesize that this effect may be modifiable. Subscapular and bicipital skinfold measurements were smaller in LTEA infants; whereas there was no significant difference between the groups with regard to suprailiac and tricipital skinfold measurements. In a previous study we have shown that in VLBW infants weight SD score at birth and disease severity were major predictors of weight SD score at TEA [8]. Altogether, these data support two important hypotheses that need to be tested in future trials. First, extrauterine growth before TEA programs future growth and body composition development. Second, a more individualized nutritional approach may be required to prevent adverse programming effects.

### 4.2. Body Composition

**Figure 1:**
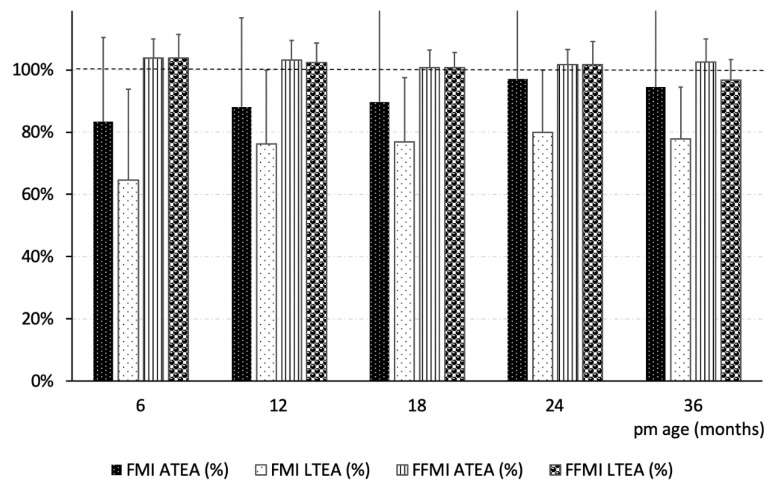
Time course of fat mass index (FMI) and fat free mass index (FFMI) in light and appropriate for term equivalent age (LTEA and ATEA) preterm infants as percentage of term infants.

This is the first longitudinal study comparing body composition development between term and preterm infants during the first 3 years of life. Contrary to our hypothesis [38], our results suggest that the smaller size of LTEA preterm infants compared those infants born at term is associated with lower FM but higher FFM when normalized for height. From 6 months corrected age to three years of life, in addition to FM also FM% and FMI were lowest in LTEA infants (group effect and interaction group x time effect both <0.05) (Figure 1). These data show that weight at term corrected age has important long-term impact on growth, suggesting an intrinsic lesser growth potential in infants born prematurely, particularly if they are light for age at 36 weeks postmenstrual age.

We report for the first time that this growth retardation has an effect on growth, body composition, and metabolic response during the first three years of life. Few previous studies only have examined and reported fat mass and lean mass during early childhood. Cooke, Rawlings, et al. studied preterm infants (*n* = 125) during the first year of life. In contrast to our infants, FM and FM% were higher than in the reference infants, wheras lean mass was reduced [39]. We speculate that in our infants improved in-hospital nutrition, and especially the protein intake close to recommended target intake, may have prevented early fat deposition. Beyond the first year of life, the number of studies comparing body composition between term and preterm infants is very limited. Darendelier et al. did not find a significant difference in fat mass index (DXA) between the preterm (about 32 weeks of gestation) and term AGA infants, or between the term and preterm SGA infants at 4–5 years of age [40]. Fewtrell et al. reported similar findings to those reported here at 8–12 years of age. The FMI and percent body fat were significantly lower in the preterm infants in contrast to FFMI [15]. Finally, Willemsen et al. found in short SGA infants at about 6.8 years significantly lower % body fat SD in preterm than in term born infants. Adjusting for gender did not change the results [41]. Hernandez et al. compared body composition between AGA and SGA VLBW preterm infants by DXA at 2 years. At 24 months, SGA VLBW infants were lighter and had less fat mass and lean mass than AGA VLBW infants [42]. Our data do not support the hypothesis that adipose tissue distribution is altered in preterm infant with significantly increased intraabdominal adiposity. There was virtually no difference with regard to truncal fat regardless of their different birth weight and birth weight z-score after appropriate adjustment for total fat mass (Table 4).

### 4.3. Bone Mineral Density

**Figure 2:**
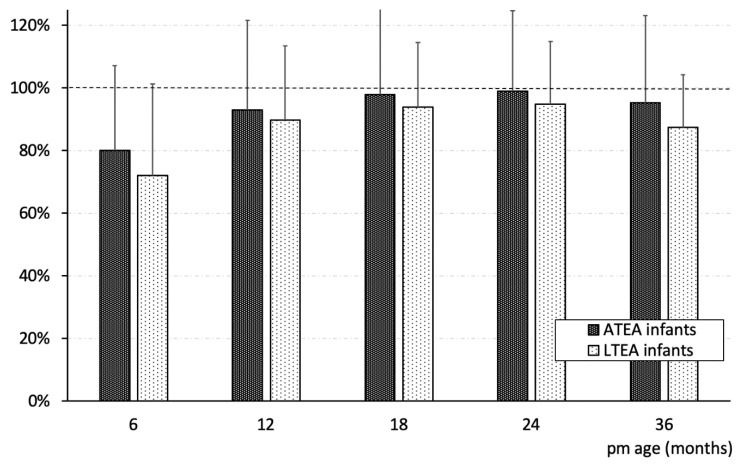
Time course of bone mineral content/length^2^ (g/mm^2^) in light and appropriate for term equivalent age (LTEA and ATEA) preterm infants as percentage of term infants. Data are given as mean ± SD.

In term infants BMC measurements at 6 and 12 months were virtually identical to published reference values [43]. Our data reconfirm studies that previously described in preterm infants a lower whole-body BMC at TEA than in infants born at full term [44,45]. At 6 months of age, in a previous study, no difference in BMC between preterm and full-term infants has been found [44], and a catch-up of BMD in preterm infants has been hypothesized [46]. In contrast, our meticulously performed longitudinal study showed a lower BMC in both groups of VLBW preterm infants throughout the first 3 years of life (Figure 2). Even after normalization for body surface area or length, there was still a significant difference between the three groups within the first 3 years of life.

For child age conflicting data have been published. One study reports a clear trend towards a reduced BMD in preterm infants, but no significant difference was found [47], whereas others report significantly reduced bone mineral density in former preterm infants when compared to term infants [48,49]. Previous reviews on bone health in adults did not indicate a significant effect of birth weight on adult BMC [50]. However, several more recent studies in adults born preterm with VLBW or SGA found lower adult bone mass, lower peak bone mass, and higher frequency of osteopenia/osteoporosis, implying an increased future fracture risk [51,52,53,54]. The most pronounced bone deficit was seen in VLBW adults [51,53]. Therefore, the reduced BMC in our preterm infants throughout the first years of life is of concern, especially in the LTEA group. The data suggest that promotion of adequate nutrition with sufficient minerals and vitamin D, and an increase in weight-bearing exercise, are thus important for former VLBW infants at all ages [53] and support the hypothesis that improved bone mineralization before discharge may improve lifelong bone health. In-hospital bone mineral accretion largely depends on enteral intake. In the present cohort, vitamin D (400 IU/d) was given in addition to standard European human milk supplements or preterm formula, and Ca, P, and vitamin D were managed on an individual basis. If 25OH-vitamin D was less than 30 ng/mL at 3 weeks of age, vitamin D supplementation was increased to 800 IU/d. If serum P was less than 5 mg/dL, infants received in addition mono-sodium-phosphate (20 mg/kg/d) and Ca-gluconate (40 mg/kg/d) given in different feedings. Therefore, current Ca and P recommendations may be insufficient to meet the needs [6,7]. We hypothesize that higher fortifier/formula mineral content or individual supplementation adjusted to urinary mineral excretion may be required [55,56].

### 4.4. Metabolic Outcome

Epidemiologic studies have described metabolic health consequences of preterm birth such as an increased risk of hypertension and insulin resistance in adult life. However, growth between birth and expected term and 12–18 months post-term has not been found to significantly effect later blood pressure or metabolic syndrome [57]. In addition, slower growth in hospital did not increase the risk of insulin resistance in the present study in preterm infants. Fasting insulin concentration and the HOMA-IR index are widely accepted measures of insulin resistance and are closely correlated with more precise but laborious measures such as an intravenous glucose-tolerance test or the euglycemic-hyperinsulinemic clamp. No differences (Table 5) were found in HOMA and fasting insulin between the three groups from 6 to 36 months of age reconfirming previous observations in preterm infants [40,58]. In addition, preterm birth and slower growth in hospital did not adversely affect hypothalamic–pituitary–adrenal axis activation. Basal morning cortisol levels were not increased in preterm infants and were lowest in preterm infants with slower in hospital growth. Long-term follow up is required to analyze whether differences in hypothalamic–pituitary–adrenal axis activation may develop later in childhood or later on [59].

It has been suggested in VLBW infants that the adverse NICU environment (especially inappropriate nutrition and increased physiological stress) during the critical early weeks ex utero leads to programmed changes in growth and in the GH-IGF-IGF binding protein axis [60]. In previous studies in VLBW infants, regardless of whether they were AGA or SGA, low plasma IGF-1 and IGFBP-3 levels were found in mid-childhood, suggesting partial GH resistance [60]. In contrast to these previous observations, in the present study, there was virtually no difference between the three groups with regard to IGFBP-1 and IGFBP-3. However, there was a significant group effect with regard to IGF-1. IGF-1 values were higher in preterm infants than in term infants at all points of time. These data suggest that the improved nutritional intake of preterm infants in hospital may have prevented the development of partial GH resistance. Long term follow-up and further analysis of the GH-IGF-IGF binding protein axis is required.

There was no significant difference between the groups with regard to leptin, sOB-R, leptin/sOB-R, adiponectin, ghrelin, and acylated ghrelin. At 6 months of age leptin/sOB-R ratio only was significantly lower in LTEA preterm infants compared to term infants. At this point of time the difference in FM% between LTEA and term infants was at a maximum. However, there was a significant difference between the groups in resistin levels. Resistin was consistently higher in LTEA infants at all times (Table 5, exception at 36 weeks). In a cross-sectional case control study of prepubertal children who were former VLBW infants with extrauterine growth retardation (appropriate for gestation at birth and less than p3 at 36 weeks), increased resistin and reduced adiponectin levels were observed [61]. Therefore, early programming of adipose tissue macrophage activation increasing the risk for later metabolic disease cannot be excluded. Further follow up of the infants is required.

### 4.5. Neurodevelopmental Outcome

Finally, our study adds to the sad growing body of evidence indicating that extremely preterm birth with very low birth weight impairs neurodevelopmental outcome. Growth is associated with neurodevelopment in VLBW preterm infants [57]. The LTEA infants presented lower head circumference in contrast to term infants. Within our target energy and protein intakes, neither energy nor protein intake but gestational age, birth weight, and illness severity were major determinants of in-hospital growth [8]. Therefore, we hypothesize that higher in-hospital target energy and protein intakes may improve in hospital growth and the neurodevelopmental outcome [62]. However, this hypothesis remains to be proven, and the safety with regard to body composition and metabolic programming needs to be evaluated as well.

### 4.6. Strengths and Limitations

The major strengths of the present study are the meticulous longitudinal assessment of children’s growth during infancy and the use of DXA to measure total and regional body composition. Thyroid dysfunction is increased in preterm infants and has been shown to impact outcome [63]. In our infants the assessment of the thyroid status was part of the clinical routine. In the present study thyroid function was investigated at the beginning of the study in every patient. It was found to be normal, and further systematic assessment of thyroid function was stopped. As in any observational study, the most important limitation is that we cannot infer causality based on the data.

## 5. Conclusions

Our results demonstrate that from six months of corrected age, and into the third year of life, gain in weight, length and head circumference, mid arm circumference, adiposity, FFM mass, and bone mineralization in preterm infants are less than in term infants and influenced by nutritional status at discharge. In hospital VLBW infants may require a higher nutrient intake. Postnatal body composition patterns indicate FFM proportional to height but lower FMI in LTEA preterm infants than term infants, with no evidence of increased truncal fat in preterm infants. Furthermore, preterm infants had poorer motor and cognitive outcomes than term infants. Longer-term consequences of this growth and body composition pattern should be studied. Since growth restriction may have permanent adverse effects, a higher in-hospital target nutrient intake is required to improve growth in these preterm infants. The clinical significance of the present lower adiposity findings is unclear. We hypothesize that this could result in a reduction in the risk of obesity. Thus, especially LTEA VLBW preterm infants would appear to benefit from targeted preventive interventions to improve growth, bone mineralization, and neurodevelopmental outcome without increasing the obesity risk. Further follow-up of our infants is required.

## Figures and Tables

**Table 1:** Anthropometric data at birth and 36 weeks postmenstrual age.

Characteristics	Patients Group	Birth	36 Weeks Postmenstrual Age
Weight (g)	Term (*n* = 34)	3299 ± 462 ^†,γ^	
	ATEA (*n* = 39)	1112 ± 214	2141± 219 *
	LTEA (*n* = 55)	1025 ± 279	1686± 245
Length (cm)	Term	49.2 ± 2.2 ^†,γ^	
	ATEA	36.0 ± 4.0	43.8 ± 1.4 *
	LTEA	35.7 ± 3.4	40.8 ± 2.3
Head circumference (cm)	Term	35.2 ± 1.6 ^†,γ^	
	ATEA	25.9 ± 2.0	31.8 ± 1.3 *
	LTEA	25.2 ± 2.7	30.3 ± 1.2
Weight z score	Term	−0.37 ± 0.93 ^†^	
	ATEA	−0.46 ± 0.49 *	−1.6 ± 0.3 *
	LTEA	−1.19 ± 0.66	−2.6 ± 0.5
Gestational age (weeks)	Term	39 ± 1 ^†,γ^	
	ATEA	28 ± 2 *	
	LTEA	29 ± 3	
Sex (male, %)	Term	68	
	ATEA	49	
	LTEA	42	
Mid arm circumference (cm)	ATEA		8.8 ± 0.5 *
	LTEA		8.0 ± 0.7

Data are expressed as mean ± SD unless stated. ATEA, appropriate for term equivalent age; LTEA, light for term equivalent age; ^†^ Term vs. LTEA < 0.05; ^γ^ Term vs. ATEA *p* < 0.05; *ATEA vs. LTEA *p* < 0.05.

**Table 2:** Basal serum values of IGF1, IGFBP1, IGFBP3, glucose, insulin, HOMA-IR, cholesterol, triglycerides, leptin, leptin receptor, adiponectin, resistin, total ghrelin, acyl ghrelin, IL-6, and cortisol at 36 weeks in LTEA and ATEA infants.

	LTEA (*n* = 45)	ATEA (*n* = 31)
IGF-1 (ng/mL)	75 ± 43	82 ± 47
IGFBP-1 (ng/mL)	37 ± 32	67 ± 113
IGFBP-3 (µg/mL)	1.45 ± 0.42	1.39 ± 0.37
Glucose (mg/dl)	81 ± 22	84 ± 19
Insulin (mcU/mL)	7 ± 5 *	11 ± 7
HOMA-IR	1.49 ± 1.33 *	2.36 ± 1.78
Cholesterol (mg/dL)	111 ± 37	101 ± 26
Triglycerides (mg/dL)	122 ± 51	95 ± 43
Leptin (ng/mL)	2.61 ± 3.08	3.05 ± 2.62
Leptin receptor (U/mL)	64 ± 27	58 ± 24
Adiponectin (µg/mL)	27 ± 11	29 ± 7
Resistin (ng/mL)	31 ± 15	29 ± 18
Total Ghrelin (pg/mL)	2122 ± 970	2011 ± 890
Acyl Ghrelin (pg/mL)	102 ± 82	98 ± 75
IL6 (pg/mL)	3.92 ± 3.56	5.21 ± 4.13
Cortisol (µg/dL)	11 ± 8	11 ± 10

Data are expressed as mean ± SD. * LTEA versus ATEA *p* < 0.05; IGF-1, insulin-like growth factor; IGFBP-1, insulin-like growth factor binding protein 1; IGFBP-3, insulin-like growth factor binding protein 3; IL6, interleukin 6; HOMA-IR, homeostatic model assessment for insulin resistance; LTEA, light for term equivalent age; ATEA, appropriate for term equivalent age.

**Table 3:** Anthropometric measurements during the first three years of life.

Anthro-Pometry	Group	6 m	12 m	18 m	24 m	36 m	*P* ^1^		
							Interaction Group * Time	Group Effect	Time Effect
Weight (g)	Term	7686 ± 1010 (34) ^†^	9770 ± 1307 (29) ^†,^^γ^	11079 ± 1406 (17) ^†,^^γ^	12553 ± 1405 (31) ^†,^^γ^	14969 ± 1925 (28) ^†,^^γ^	<0.01	<0.001	<0.001
	ATEA	7299 ± 823 (31) *	8768 ± 1026 (28) *	9999 ± 1266 (17)	11553 ± 1336 (28)	13260 ± 1645 (20)			
	LTEA	6306 ± 979 (51)	8085 ± 1213 (43)	9474 ± 1201 (40)	10572 ± 1493 (46)	12117 ± 1640 (41)			
Length (cm)	Term	68.0 ± 2.7 ^†^	76.1 ± 2.3 ^†,^^γ^	84 ± 4 ^†,^^γ^	89 ± 4 ^†,^^γ^	97 ± 4 ^†,^^γ^	NS	<0.001	<0.001
	ATEA	66.5 ± 3.1 *	73.7 ± 2.6	80 ± 3	87 ± 4 *	93 ± 4			
	LTEA	63.5 ± 3.2	72.1 ± 3.1	79 ± 3	84 ± 4	91 ± 4			
HC (cm)	Term	44.2 ± 1.7 ^†^	47.2 ± 1.6 ^†^	48.8 ± 1.6 ^†^	49.6 ± 1.8 ^†^	50.8 ± 1.8 ^†,^^γ^	NS	<0.001	<0.001
	ATEA	43.7 ± 1.2 *	46.3 ± 1.2 *	47.9 ± 1.3	48.9 ± 1.4 *	49.7 ± 1.1 *			
	LTEA	42.5 ± 1.8	45.2 ± 1.8	46.7 ± 1.5	47.5 ± 1.7	48.4 ± 1.8			
Mid arm circum-ference (cm)	Term	14.5 ± 1.1(34) ^†^	15.4 ± 1.4 (28) ^†^	15.3 ± 1.4 (17)	16.4 ± 1.2 (30) ^†^	16.9 ± 1.4 (28) ^†^	<0.01	<0.001	<0.001
	ATEA	14.4 ± 0.9 (29) *	14.7 ± 1.1 (28)	15.4 ± 1.3 (17)	15.8 ± 1.3 (22)	16.3 ± 1.1 (18)			
	LTEA	13.2 ± 1.3 (50)	14.1 ± 1.2 (43)	14.8 ± 1.2 (39)	15.2 ± 1.0 (40)	15.5 ± 1.1 (38)			
Sub-scapular skinfold (cm)	Term	7.1 ± 1.5(34)	6.8 ± 1.3 (29)	6.3 ± 1.5 (17)	6.3 ± 1.4 (30)	6.2 ± 1.4 (28)	NS	<0.001	<0.001
	ATEA	6.9± 1.4 (31)	6.3 ± 1.3 (27)	6.1 ± 1.3 (17)	5.8 ± 1.3 (22)	5.6 ± 1.4 (17)			
	LTEA	6.2 ± 1.4 (50)	5.6 ± 1.0 (43)	5.5 ± 1.2 (39)	5.3 ± 1.0 (39)	5.1 ±1.0 (38)			
Tricipital skinfold (cm)	Term	7.9 ± 1.9(34) ^†^	7.8 ± 2.5 (29) ^†^	6.3 ± 1.5 (17)	8.2 ± 1.8 (30)	8.6 ± 1.9 (28) ^†,^^γ^	<0.01	NS	<0.001
	ATEA	8.2 ± 1.8 (31) *	7.3 ± 1.6 (27)	6.8 ± 1.3 (17)	7.5 ± 1.5 (22)	7.3 ± 1.3 (17)			
	LTEA	6.8 ± 1.3 (50)	6.7 ± 1.5 (43)	6.9 ± 1.2 (39)	7.5 ± 1.5 (39)	7.3 ±1.5 (38)			
Bicipital skinfold (cm)	Term	6.8 ± 1.7(34) ^†^	5.8 ± 1.6 (29)	5.6 ± 1.6 (16)	5.8 ± 1.5 (30)	5.8 ± 1.3 (28)	NS	<0.01	<0.001
	ATEA	6.3 ± 1.2 (31)	5.8 ± 1.7 (27)	5.4 ± 1.6 (17)	5.4 ± 1.1 (22)	5.3 ± 1.3 (17)			
	LTEA	5.6 ± 1.2 (50)	5.3 ± 1.4 (43)	5.1 ± 1.1 (39)	5.4 ± 1.1 (39)	5.2 ±1.1 (37)			
Suprailiac skinfold (cm)	Term	7.1 ± 1.8(34)	6.3 ± 1.9 (29)	5.2 ± 1.2(15)	6.4 ± 1.7 (30)	5.8 ± 1.9 (28)	NS	NS	<0.001
	ATEA	7.4 ± 1.9 (31)	6.2 ± 1.8 (27)	6.7 ± 2.3 (17)	6.3 ± 1.9 (22)	6.6 ± 2.1 (17)			
	LTEA	6.7 ± 1.8 (50)	5.9 ± 1.7 (42)	6.0 ± 1.9 (39)	6.0 ± 1.4 (39)	6.1 ±1.9 (38)			

Data are express as mean ± SD unless stated. ATEA, appropriate for term equivalent age; LTEA, light for term equivalent age; HC, head circumference; m, months; NS, not significant; ^†^ Term vs. LTEA < 0.05. ^γ^ Term vs. ATEA *p* < 0.05. * ATEA vs. LTEA *p* < 0.05. ^1^ Linear mixed model.

**Table 4:** Body composition and bone densitometry results (by dual-energy X-ray absorptiometry).

	Group	6 m	12 m	18 m	24 m	36 m	*P* ^1^		
							Interaction Group * Time	Group Effect	Time Effect
FM (g)	Term	2234 ± 681(22) ^†,^^γ^	2446 ± 865(25) ^†^	2756 ± 854(15) ^†^	2787 ± 840 (24) ^†^	3407 ± 1036(25) ^†^	<0.05	<0.001	<0.001
	ATEA	1813 ± 625(26) *	2008 ± 681(25)	2241 ± 809(17)	2591 ± 667(14)	2949 ± 920(13)			
	LTEA	1282 ± 597(46)	1660 ± 580(43)	1877 ± 589(34)	2045 ± 580(35)	2323 ± 588(34)			
FFM (g)	Term	6059 ± 729 ^†^	7451 ± 939 ^†^	8939 ± 795	9581 ± 839 ^†^	11517 ± 1234 ^†^	<0.05	<0.001	<0.001
	ATEA	5982 ± 517 *	7117 ± 642	8049 ± 952	9221 ± 876	10891 ± 1349			
	LTEA	5495 ± 675	6832 ± 844	7880 ± 790	8824 ± 978	9796 ± 1213			
FM (%)	Term	27 ± 5(22) ^†,^^γ^	24 ± 5(25) ^†^	23 ± 6(15)	22 ± 5(24) ^†^	22 ± 4(25)	<0.01	<0.01	NS
	ATEA	23 ± 6(26)	22 ± 6(25)	21 ± 6(17)	22 ± 4(14)	21 ± 5(13)			
	LTEA	18 ± 6(46)	19 ± 5(43)	19 ± 4(34)	19 ± 4(35)	19 ± 4(34)			
FFM (%)	Term	74 ± 6 ^†,^^γ^	76 ± 5 ^†^	77 ± 6	78 ± 5 ^†^	78 ± 4	<0.05	<0.01	NS
	ATEA	77 ± 6(26)	78 ± 6	79 ± 6	78 ± 4	79 ± 5			
	LTEA	82 ± 6(46)	81 ± 5	81 ± 4	81 ± 4	81 ± 4			
FMI (g/cm^2^)	Term	0.48 ± 0.13 ^†,^^γ^	0.42 ± 0.13 ^†^	0.39 ± 0.11	0.35 ± 0.09	0.36 ± 0.09	<0.05	<0.01	<0.001
	ATEA	0.40 ± 0.13	0.37 ± 0.12	0.35 ± 0.12	0.34 ± 0.09	0.34 ± 0.10			
	LTEA	0.31 ± 0.14	0.32 ± 0.10	0.30 ± 0.08	0.28 ± 0.07	0.28 ± 0.06			
FFMI (g/cm^2^)	Term	1.31 ± 0.12 (22)	1.28 ± 0.13 (25)	1.26 ± 0.05 (15)	1.21 ± 0.05 (24)	1.21 ± 0.08 (24)	NS	NS	<0.001
	ATEA	1.36 ± 0.08 (25)	1.32 ± 0.08 (25)	1.27 ± 0.07 (16)	1.23 ± 0.06 (14)	1.24 ± 0.09 (13)			
	LTEA	1.36 ± 0.10 (46)	1,31 ± 0.08 (43)	1.27 ± 0.06 (34)	1.23 ± 0.09 (35)	1.17 ± 0.08 (34)			
BMD (g/cm^2^)	Term	0.57 ± 0.05(30) ^†,^^γ^	0.63 ± 0.04(25)	0.69 ± 0.05(15) ^†^	0.71 ± 0.05(24)	0.77 ± 0.05(25)	NS	<0.001	<0.001
	ATEA	0.54 ± 0.05(27)	0.60 ± 0.04(25)	0.67 ± 0.05(17)	0.70 ± 0.03(14)	0.75 ± 0.04(13)			
	LTEA	0.52 ± 0.05(46)	0.60 ± 0.04(43)	0.65 ± 0.06(33)	0.69 ± 0.04(35)	0.74 ± 0.04(34)			
BMC (g)	Term	167 ± 39 ^†,^^γ^	244 ± 46 ^†^	339 ± 55 ^†,^^γ^	401 ± 73 ^†,^^γ^	547 ± 89 ^†,^^γ^	<0.001	<0.001	<0.001
	ATEA	129 ± 31	210 ± 41	293 ± 67	377 ± 53	482 ± 64			
	LTEA	106 ± 29	196 ± 44	281 ± 55	345 ± 56	421 ± 65			
BMC/length^2^ (g/mm^2^)	Term	3.62 ± 0.78 ^†,^^γ^(30)	4.16 ± 0.66 (25)	4.79 ± 0.54 (15)	5.05 ± 0.59 (24)	5.76 ± 0.68 ^†^ (24)	<0.001	<0.001	<0.001
	ATEA	2.90 ± 0.66 (27)	3.87 ± 0.59 (25)	4.69 ± 0.65 (16)	5.00 ± 0.54 (14)	5.49 ± 0.53 (13)			
	LTEA	2.61 ± 0.62 (46)	3.73 ± 0.66 (43)	4.50 ± 0.68 (34)	4.79 ± 0.64 (35)	5.04 ± 0.55 (34)			
TF (g)	Term	681 ± 230 (30) ^†,^^γ^	683 ± 368 (25) ^†^	768 ± 300(15) ^†^	728 ± 288 (24) ^†^	984 ± 370 (25) ^†^	<0.01	<0.001	<0.001
	ATEA	512 ± 237 (27)	538 ± 251 (25)	604 ± 312 (17)	674 ± 229 (14)	865 ± 378 (13)			
	LTEA	358 ± 186 (46)	440 ± 199 (43)	480 ± 219(34)	519 ± 209 (35)	680 ± 250(34)			
PTF (%)	Term	28 ± 3	26 ± 6	27 ± 3	26 ± 3	28 ± 4	NS	NS	<0.001
	ATEA	27 ± 4	26 ± 6	26 ± 5	26 ± 3	29 ± 4			
	LTEA	28 ± 5	26 ± 4	25 ± 4	25 ± 4	29 ± 5			

Data are expressed as mean ± SD unless stated. ATEA, appropriate for term equivalent age; LTEA, light for term equivalent age; BMC, bone mineral content; BMD, bone mineral density; FFM, fat free mass; FFMI, free fat mass index; FM, fat mass; FMI, fat mass index; PTF, percentage of trunk fat mass; TF, trunk fat; ^†^ Term vs. LTEA < 0.05; ^γ^ Term vs. ATEA *p* < 0.05; * ATEA vs. LTEA *p* < 0.05; ^1^ Linear mixed model.

**Table 5:** Comparison of cholesterol and triglycerides, adipokines, interleukin 6, IGF family, glucose tolerance parameters, and cortisol between term, LTEA, and ATEA infants.

	Patient Group	6 m	N	12 m	N	18 Months	N	24 Months	N	36 Months	N	P ^1^		
												Interaction Group * Time	Group Effect	Time Effect
IGF-1 (ng/mL)	Term	94 ± 48	34	94 ± 52	26	59 ± 22	16	67 ± 39	29	78 ± 30	29	NS	<0.05	NS
	ATEA	108 ± 55	29	117 ± 101	26	88 ± 70	14	95 ± 57	18	88 ± 46	17			
	LTEA	103 ± 67	48	114 ± 83	40	91 ± 43	35	88 ± 46	37	87 ± 34	40			
IGFBP-1 (ng/mL)	Term	52 ± 36	34	65 ± 30 ^γ^	26	43 ± 24	15	43 ± 14	19			NS	NS	<0.05
	ATEA	50 ± 28	29	51 ± 25	23	41 ± 12	6	58 ± 30	4					
	LTEA	49 ± 29	49	55 ± 31	37	45 ± 17	18	37 ± 6	7					
IGFBP-3 (µg/mL)	T	2.41 ± 0.5	34	2.56 ± 0.71	25	2.39 ± 0.82	17	2.35 ± 0.80	28	2.61 ± 0.51	29	NS	NS	<0.01
	ATEA	2.29 ± 0.51	29	2.38 ± 0.72	25	2.18 ± 0.91	15	2.37 ± 0.80	18	2.70 ± 0.61	17			
	LTEA	2.37 ± 0.71	50	2.58 ± 0.54	36	2.43 ± 0.9	37	2.25 ± 0.82	41	2.63 ± 0.69	39			
Plasma glucose (mg/dL)	T	77 ± 12	8	75 ± 8	26	73 ± 9	17	76 ± 6	29	80 ± 5	29	NS	NS	<0.01
	ATEA	80 ± 6	16	76 ± 5	25	75 ± 7	14	77 ± 7	18	78 ± 13	17			
	LTEA	77 ± 7	31	74 ± 8	41	76 ± 9	40	79 ± 8	43	79 ± 7	41			
Insulin (mcU/mL)	T	2.64 ± 1.28	34	2.51 ± 1.24	28	2.44 ± 1.43	17	2.60 ± 1.07	29	3.85 ± 1.70	29	NS	NS	<0.001
	ATEA	2.99 ± 1.69	27	2.56 ± 1.33	26	2.58 ± 1.24	15	3.45 ± 1.86	18	3.70 ± 1.64	17			
	LTEA	3.00 ± 1.62	46	2.66 ± 1.53	39	2.84 ± 1.44	36	3.79 ± 1.77	39	3.49 ± 2.12	40			
HOMA	Term	0.5085 ± 0.195	8	0.469 ± 0.244	25	0.454 ± 0.319	17	0.492 ± 0.220	29	0.775 ± 0.370	29	NS	NS	<0.001
	ATEA	0.680 ± 0.421	14	0.497 ± 0.288	24	0.488 ± 0.253	14	0.673 ± 0.395	18	0.768 ± 0.390	16			
	LTEA	0.604 ± 0.338	26	0.493 ± 0.319	37	0.556 ± 0.329	36	0.746 ± 0.398	39	0.695 ± 0.451	40			
Cholesterol (mg/dL)	Term	132 ± 33	26	146 ± 25	28	151 ± 28	17	152 ± 27	29	150 ± 27	28	NS	NS	<0.001
	ATEA	138 ± 25	27	144 ± 38	26	159 ± 38	15	159 ± 32	18	158 ± 32	17			
	LTEA	135 ± 24	46	159 ± 29	41	158 ± 28	40	160 ± 35	42	165 ± 30	38			
Triglycerides (mg/dL)	Term	105 ± 33	25	116 ± 51	27	81 ± 37	17	70 ± 16	29	68 ± 22	28	NS	NS	<0.001
	ATEA	116 ± 41	27	108 ± 41	26	104 ± 61	15	78 ± 26	18	72 ± 26	17			
	LTEA	114 ± 45	44	107 ± 47	41	87 ± 32	40	79 ± 29	42	67 ± 19	38			
Leptin (ng/mL)	T	5.60 ± 2.74 ^†^	34	4.40 ± 4.38	29	2.87 ± 1.21	17	3.30 ± 1.71	29	3.49 ± 1.52	29	<0.05	NS	<0.001
	ATEA	6.16 ± 3.54 *	30	4.31 ± 3.01 *	27	3.75 ± 1.95	15	3.53 ± 1.84	18	4.32 ± 2.69	17			
	LTEA	4.21 ± 1.80	50	3.18 ± 1.59	43	3.40 ± 1.41	39	3.60 ± 1.56	42	3.28 ± 1.54	40			
sOB-R (U/mL)	Term	66 ± 21	34	72 ± 30	28	66 ± 17	17	72 ± 19	29	50 ± 13	29	NS	NS	<0.001
	ATEA	70 ± 21	30	76 ± 27	25	76 ± 20	14	66 ± 21	18	54 ± 17	17			
	LTEA	69 ± 23	51	79 ± 20	42	76 ± 23	37	68 ± 19	42	57 ± 13	40			
Leptin/sOB-R	Term	0.12 ± 0.18 ^†^	34	0.08 ± 0.06 ^†^	28	0.05 ± 0.02	17	0.05 ± 0.03	29	0.08 ± 0.06	29	NS	NS	<0.001
	ATEA	0.11 ± 0.12	31	0.08 ± 0.13	25	0.06 ± 0.04	15	0.07 ± 0.06	19	0.11 ± 0.13	18			
	LTEA	0.07 ± 0.05	49	0.05 ± 0.04	41	0.06 ± 0.06	36	0.06 ± 0.03	41	0.06 ± 0.04	39			
Adiponectin (µg/mL)	Term	27 ± 9	32	23 ± 6	28	28 ± 11	17	25 ± 8	29	21 ± 7	28	<0.05	NS	<0.05
	ATEA	25 ± 9	30	24 ± 9	26	22 ± 6	15	20 ± 5	18	20 ± 5	17			
	LTEA	26 ± 7	50	24 ± 9	44	22 ± 7	37	24 ± 8	42	25 ± 9	40			
Resistin (ng/mL)	T	15 ± 10	34	19 ± 8	25	22 ± 9	15	18 ± 9	27	18 ± 8	23	NS	<0.05	<0.05
	ATEA	17 ± 12	29	17 ± 6	25	17 ± 11	13	19 ± 10	16	19 ± 7	15			
	LTEA	23 ± 16	50	24 ± 12	41	19 ± 9	39	20 ± 10	40	21 ± 11	35			
Total Ghrelin (pg/mL)	T	3107 ± 1951	34	2977 ± 1120	28	2805 ± 1702	17	2406 ± 778	29	2055 ± 702	29	NS	NS	<0.001
	ATEA	3465 ± 1739	30	3414 ± 1342	27	2563 ± 991	15	2166 ± 868	18	1923 ± 658	17			
	LTEA	3222 ± 1385	50	3234 ± 1215	44	2947 ± 1559	39	2343 ± 1050	42	2354 ± 1226	39			
Ghrelin acylate (pg/mL)	T	95 ± 51	34	85 ± 39	28	62 ± 24	17	62 ± 24	29	43 ± 31	29	NS	NS	<0.001
	ATEA	82 ± 47	29	79 ± 52	27	64 ± 49	15	58 ± 27	18	44 ± 29	17			
	LTEA	102 ± 64	51	66 ± 36	43	57 ± 27	39	53 ± 23	40	47 ± 35	40			
IL6 (pg/mL)	T	2.77 ± 3.5	31	3.01 ± 2.96	21	2.25 ± 1.29	7	1.82 ± 1.19	14			NS	<0.05	NS
	ATEA	1.08 ± 0.83	21	1.73 ± 1.23	17	0.97 ± 0.45	7	1.48 ± 1.76	6					
	LTEA	2.45 ± 2.14	40	3.11 ± 2.97	33	1.88 ± 1.74	23	2.85 ± 3.16	11					
Cortisol (µg/dL)	T	13 ± 5	28	12 ± 4	29	10 ± 2	17	10 ± 4	29	9 ± 3	28	NS	<0.01	<0.01
	ATEA	11 ± 6	27	11 ± 6	27	14 ± 4	15	11 ± 3	18	8 ± 2	16			
	LTEA	9 ± 5	49	10 ± 5	42	9 ± 4	39	9 ± 4	41	8 ± 2	33			

Data are expressed as mean ± SD unless stated. T, term; ATEA, appropriate for term equivalent age; LTEA, light for term equivalent age; M, months; N, number; ^†^ Term vs. LTEA < 0.05; ^γ^ Term vs. ATEA *p* < 0.05; * ATEA vs. LTEA *p* < 0.05; ^1^ Linear mixed model.

## Data Availability

The data presented in this study are available on request from the corresponding author. The data are not publicly available due to ethical reasons.
